# User Acceptance of Remote Care Assist, a Telecare System for Home Care Among Care and Nursing Staff: Cross-Sectional Pilot Study

**DOI:** 10.2196/80514

**Published:** 2026-06-03

**Authors:** Friedrich Ebner, Ulrike Schneider, Cornelia Schneider, Birgit Trukeschitz

**Affiliations:** 1 Research Institute for Economics of Aging, WU Vienna University of Economics and Business, Welthandelsplatz 1, Building D5, Vienna, 1020, Austria, 43 1 31336 5398; 2 Department of Socioeconomics, Institute for Social Policy, Research Institute for Economics of Aging, WU Vienna University of Economics and Business, Vienna, Austria; 3 Department of Information Technologies and Digitalisation, Salzburg University of Applied Sciences, Salzburg, Austria

**Keywords:** home care services, nurse-patient relation, nursing staff, remote consultation, user acceptance, videoconferencing

## Abstract

**Background:**

Demographic and epidemiological changes are increasing pressure on health and long-term care systems, underscoring the need for digital innovations. Remote Care Assist is a digital system that enables home care staff to connect with care experts for exchange and support via real-time video calls. Although technology acceptance is crucial for successful implementation, little is known about how care staff’s expected benefits for care recipients influence acceptance in professional home care.

**Objective:**

This study examined predictors of user acceptance of the Remote Care Assist among home care staff, with a particular focus on the role of staff’s expectations of benefits for home care service users.

**Methods:**

Technology acceptance data were collected from staff in home care organizations in Austria and Luxembourg. Among 337 survey respondents, 139 participants who reported using Remote Care Assist at least once per month over a period of 5-6.5 months were included in the acceptance analysis (45 care experts and 94 on-site care staff). Partial least squares structural equation modeling was used to test a contextualized technology acceptance model.

**Results:**

Technology acceptance was measured by “Behavioral Intention to Use” the Remote Care Assist. “Behavioral Intention to Use” was positively associated with “Expected Benefit for Home Care Service Users” (EBC; *β*=0.506, 95% CI 0.364 to 0.658; *P*<.001), “Perceived Usefulness (PU)” for care staff (*β*=0.314, 95% CI 0.151 to 0.460; *P*<.001), and “Perceived Ease of Use” (PEOU; *β*=0.130, 95% CI 0.038 to 0.231; *P*=.01). “EBC” (*β*=0.415, 95% CI 0.276 to 0.537; *P*<.001), “Perceived Efficiency” (*β*=0.396, 95% CI 0.267 to 0.531; *P*<.001), and “PEOU” (*β*=0.170, 95% CI 0.083 to 0.266; *P*=.001) were positively associated with “PU” for care staff. “PU” also positively mediated the associations of “EBC” (*β*=0.130, 95% CI 0.061 to 0.194; *P*=.001) and “PEOU” (*β*=0.053, 95% CI 0.017 to 0.101; *P*=.02) with “Behavioral Intention to Use.” “Reliable Functionality” was not significantly associated with “PU.”

**Conclusions:**

This study suggests that the technology acceptance of a digital system for enhancing professional exchange between different staff groups in home care is shaped not only by established predictors of acceptance, such as PU and PEOU, but also by a currently neglected predictor, namely care staff’s expectations that the technology will benefit home care service users, which plays an important role in technology acceptance. In addition to usability and workflow support, successful implementation strategies for digital technologies should clearly communicate the technology’s potential benefits for care staff, care service users, and the broader care ecosystem.

## Introduction

### Background

The rapid proliferation of digital communication technologies has transformed a number of sectors, with the COVID-19 pandemic accelerating this trend [1]. Although the health care sector is no exception, it has been slower to adopt digital solutions compared to other sectors, such as banking and general insurance [2]. Electronic documentation, for example, has been rolled out later and more slowly [3]. Even within the health care sector, the adoption of digital technologies varies greatly, with long-term care lagging further behind than acute care. In particular, implementing new digital apps in home care settings poses challenges, such as limited time and financial resources [4,5], concerns regarding data security and privacy [6], and resistance or lack of acceptance among care staff. These barriers underscore the importance of designing and evaluating telecare technologies that fit the needs of home care practice.

### Remote Care Assist

Against this background, research and development projects cofunded by national and European funding agencies aim to develop tailored digital solutions for home care settings. One of these projects, titled “Care about Care,” developed and tested a communication system to support care workers in the homes of people receiving care. This so-called “Remote Care Assist” system enabled highly qualified and more experienced care staff (henceforth “care experts”) to provide support and advice via video calls [7]. To this end, Remote Care Assist consisted of 2 components. The first component was a “remote support app” on care and nursing staff’s Android and iOS company phones to enable video streaming between members of the care team. In addition, care experts also had access to a web app called “the expert center” to receive and make such calls in their offices using laptops or stationary computers. The system enabled care experts to see what their colleagues see in their clients’ homes. Different tools supported them in interactive tasks, such as marking and measuring specific wound regions on the screen. In addition, care experts could save screenshots as part of the care documentation. This digital technology thus aimed to facilitate communication and decisions on the appropriate management of wounds, medication, and other care and nursing issues.

### Technology Acceptance

For technologies such as Remote Care Assist, successful implementation depends not only on technical performance, but also on end-user acceptance. Acceptance research aims to identify predictors that positively or negatively influence end users’ behavioral intention to use a product or service [8]. Because acceptance determinants can vary across contexts, technology acceptance models (TAMs) should be validated for specific contexts, technologies, and end-user groups [9,10].

Established models such as the TAM [11] and the Unified Theory of Acceptance and Use of Technology (UTAUT) [12] are regarded as the gold standard in acceptance research across various apps and industries, including the health care sector [9].

### Analytical Model and Hypotheses

**Figure 1.**
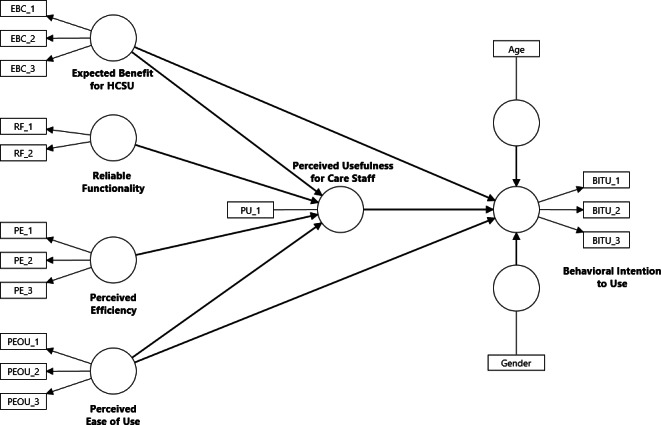
Proposed technology acceptance model. EBC_1-3: measurement items for “Expected Benefit for HCSU,” RF_1-2: measurement items for “Reliable Functionality,” PE_1-3: measurement items for “Perceived Efficiency,” PEOU_1-3: measurement items for “Perceived Ease of Use,” PU_1: measurement item for “Perceived Usefulness for Care Staff,” and BITU_1-3: measurement items for “Behavioral Intention to Use.” HCSU: home care service user.

TAMs have been widely adopted and further developed in previous studies [13-20]. Building on this body of research, we propose a contextualized model (Figure 1) in which care staff’s “Behavioral Intention to Use” Remote Care Assist is associated with “Perceived Usefulness (PU)” and “Perceived Ease of Use (PEOU).” We extend the model by incorporating “Expected Benefit for Home Care Service Users (EBC)” and by modeling “Perceived Efficiency (PE)” and “Reliable Functionality (RF)” as antecedents of “PU.”

“PU” has emerged as one of the most influential factors in acceptance research [10,21-23]. The predictor is described as the degree to which an individual believes that using the system will help him or her to attain gains in job performance [12]. We hypothesized a direct association of “PU” for care staff with “Behavioral Intention to Use” Remote Care Assist. Hypothesis 1 was that “PU” for care staff is positively associated with the “Behavioral Intention to Use” Remote Care Assist among care staff.

“EBC” reflects the degree to which care staff believe that home care service users will benefit from the use of the system. We hypothesized a dual motivation for technology acceptance in long-term care settings, which consists of both a direct association of “EBC” with “Behavioral Intention to Use” Remote Care Assist and an indirect mediated relationship through “PU” for care staff. The mediation suggests that care staff who expect digital care technology to be beneficial for their care recipients are more likely to perceive it as useful, which, in turn, enhances their future “Behavioral Intention to Use” the Remote Care Assist. Hypothesis 2a was that “EBC” is positively associated with “Behavioral Intention to Use” Remote Care Assist among care staff, Hypothesis 2b was that “EBC” is positively associated with “PU” for care staff, and Hypothesis 2c was that the relationship between “EBC” and “Behavioral Intention to Use” Remote Care Assist among care staff is positively mediated by “PU” among care staff. While “PU” in acceptance research [12] typically emphasizes efficiency improvement, our study proposed “PU” among care staff as an attitude, with “PE” and “RF” as direct antecedents. “PE” is defined as the degree to which the system will increase the productivity of care staff. “RF” is related to “Output Quality” [8,20]. However, because telecare technologies are not primarily output-oriented, we defined “RF” as the degree to which the system is considered to work consistently and error-free in everyday professional use. Hypothesis 3 was that “PE” is positively associated with “PU” among care staff, and Hypothesis 4 was that “RF” is positively associated with “PU” among care staff.

Consistent with previous studies [24,25], “PEOU” often emerges as an important predictor of “Behavioral Intention to Use,” proposing that end users who perceive information technology as easy to use are more likely to accept it for further use. Therefore, “PEOU” is defined as the degree of ease associated with consumers’ use of technology [19]. We hypothesized a direct association of “PEOU” with the “Behavioral Intention to Use“ Remote Care Assist and an indirect mediated relationship through “PU” for care staff. The hypothesized mediation suggests that care staff who perceive telecare technology as user-friendly are more likely to perceive it as useful, which in turn enhances their future “Behavioral Intention to Use” Remote Care Assist. Hypothesis 5a was that “PEOU” is positively associated with “Behavioral Intention to Use” Remote Care Assist among care staff, Hypothesis 5b was that “PEOU” is positively associated with “PU” for care staff, and Hypothesis 5c was that the relationship between the “PEOU” and “Behavioral Intention to Use” Remote Care Assist among care staff is positively mediated by “PU.”

“Behavioral Intention to Use” Remote Care Assist is defined as the care staff’s subjective probability of using the system in the future [26]. In accordance with previous acceptance studies [13-15,27,28], this construct was designated as the principal dependent variable of this study. This choice was justified by the fact that Remote Care Assist was examined in a pilot context and was therefore not available on the open market at the time of the study.

### Objective

The objective of this study was to test a contextualized TAM for the “Remote Care Assist” system in professional home care. Specifically, we examined the established associations of “PU” for care staff and “PEOU” with “Behavioral Intention to Use” Remote Care Assist. Additionally, we evaluated “PE” and “RF” as antecedents of “PU.” In professional care contexts, however, adoption decisions may also reflect considerations beyond the individual user’s own efficiency, including whether a technology is expected to benefit care recipients. While prior research has examined “Perceived Customer Usefulness” in social work contexts [29], little is known about how care staff’s expectations of benefits for home care service users influence their intention to use telecare in routine professional practice. This study addresses this gap by extending established acceptance predictors with the EBC and by exploring system- and workflow-related antecedents of the behavioral intention to use a newly developed telecare application.

## Methods

### Study Design and Participants

The Remote Care Assist was tested in 2 home care organizations in Austria and Luxembourg [30]. Study participants were recruited by the participating care organizations using a 2-stage sampling procedure. First, all care units within each organization were screened for eligibility. Care units were eligible if they did not have major staffing shortages and did not participate in pretests of the technology. Second, all employees of eligible care units were considered eligible for participation. Participants were classified according to their professional role as care experts (registered nurses or the unit’s care managers) or care and nursing staff (nursing assistants and home helpers). All participants were trained in the use of the system (mobile app and expert center) using a peer-to-peer training approach. For more details, refer to the study [30].

Approximately half of the eligible care units were randomly assigned to the test group. Care workers in test-group care units received access to the Remote Care Assist app on their company smartphones. Care experts had access to the web-based care expert center within their assigned units. They did not provide support across units. The acceptance analysis focuses on exposed users rather than on comparisons between randomized groups. Only participants who reported using Remote Care Assist at least once per month were included in the acceptance analyses. To ensure transparency and complete reporting, we followed the CHERRIES (Checklist for Reporting Results of Internet E-Surveys checklist [Checklist 1]).

### Sample Size

Sample size considerations were guided by the 10-times rule for partial least squares structural equation modeling (PLS-SEM) based on the maximum number of direct incoming paths to any endogenous construct [31]. In our structural model, “Behavioral Intention to Use” was the endogenous construct with the largest number of direct predictors: “EBC,” “PU” for care staff, “PEOU,” and the control variables age and gender. Therefore, we determined the minimum required sample size to be 50 valid responses. To assess the adequacy of the achieved sample after the survey, we additionally conducted a PLS-SEM power analysis in line with established recommendations [32].

### Data Collection

The acceptance survey was conducted as a special topic survey and as part of the second wave of the study’s data collection. The original questionnaire was developed in German and translated into French for French-speaking participants in Luxembourg. Before administration, both language versions were pretested in Austria and Luxembourg. Eligible participants were informed about the project by their colleagues participating in the project consortium. The survey was administered to all eligible care and nursing staff (care workers and nursing assistants) and care experts (registered nurses, wound managers, and care managers). The link provided in a text message directed them to a language-specific LimeSurvey (LimeSurvey GmbH) questionnaire. It comprised modules with questions on potential outcomes of the technology, usability, usage behavior, and technology acceptance. The survey included 65 questions for care staff and 74 questions for care experts. Responses were recorded automatically. Participants could discontinue the survey and resume it at any time using their individual token. Trial periods lasted from March to September 2023, with survey data collection from September 13 to October 15, 2023, in Austria and from May to October 2023, with survey data collection from September 14 to October 30, 2023, in Luxembourg. Differences in the data collection period resulted from a delay in ethical approval in Luxembourg.

### Measures

The technology acceptance section of the online survey included 15 items adapted from established acceptance research [11,12] and tailored to the Remote Care Assist context. Construct definitions, item wordings, and variable names were adapted from prior validated instruments, particularly the UTAUT [12], and tailored to the technology used in home care settings (Multimedia Appendix 1).

The outcome variable, “Behavioral Intention to Use” Remote Care Assist, reflected respondents’ willingness to use the system in the future and represented technology acceptance. It was assessed using 3 items (BITU_1 to BITU_3). “PU” for care staff, specified as a mediator, captured the perceived benefits for care and nursing staff and was measured using a single global item (PU_1). This was in line with prior research using parsimonious measures [33,34]. The remaining predictors were operationalized as follows: EBC_1 to EBC_3, PE_1 to PE_3, RF_1 to RF_2, and PEOU_1 to PEOU_3. All acceptance items were rated on a 6-point Likert-type agreement scale ranging from 1 (“strongly disagree”) to 6 (“strongly agree”). An even-numbered response format was used to reduce midpoint responding, as midpoint selections may indicate forced neutrality [35], serve as a reference point [36], or fail to reflect respondents’ true opinions [37].

In addition, consistent with previous studies on technology acceptance [12,25,38,39], age and gender were included as covariates to control for potential differences related to the “Behavioral Intention to Use” the Remote Care Assist. Age was dichotomized using two groups: 20-45 years (lower-age group=0) and 46-60 years (higher-age group=1). Gender was coded as women=0 and men=1.

### Statistical Analysis

Descriptive statistics were conducted using R (R Foundation for Statistical Computing). Technology acceptance data were analyzed using SmartPLS (version 4; SmartPLS GmbH). The proposed model was analyzed using PLS-SEM, enabling simultaneous assessment of the measurement model and testing of the hypothesized structural relationships. This approach is particularly suited for exploratory research with small sample sizes [40]. It is a nonparametric method and therefore does not require any distributional assumptions. We used statistical bootstrapping to provide more accurate SEs and CIs and thus reliable parameter estimation [31].

First, we evaluated the measurement models by examining their reliability and validity, with a focus on internal consistency reliability, indicator reliability, convergent validity, and discriminant validity [40]. Internal consistency was evaluated by Cronbach alpha and composite reliability (RhoA and RhoC [41]). Indicator reliability was evaluated based on indicator loadings associated with the underlying constructs. Convergent validity was evaluated using the average variance extracted. Discriminant validity was assessed using the Heterotrait-Monotrait ratio of correlations (HTMT).

After confirming the validity and reliability of the measurement models, we evaluated the hypothesized structural model [40]. This included assessing collinearity among predictor constructs using variance inflation factors (VIFs), estimating the magnitude and significance of the structural path coefficients, and examining the model’s explanatory and predictive power. We applied the PLS-SEM bootstrapping procedure using 10,000 subsamples and assessed directional hypotheses using one-tailed tests at the 5% level. In addition, we report bootstrap CIs. Further, we conducted a mediation analysis to test the hypothesized direct and indirect mediations.

Additionally, we performed an importance-performance map analysis [42] to visualize and classify predictors into 4 quadrants according to their relative importance and performance on “Behavioral intention to Use” Remote Care Assist. The importance threshold was defined as the mean total effect across the key constructs, and the performance threshold was defined as the grand mean of the corresponding construct mean scores. Based on these thresholds, constructs were assigned to 1 of 4 quadrants: low importance and low performance, low importance and high performance, high importance and low performance, and high importance and high performance. The positions of each predictor in the matrix were visualized to identify potential intervention priorities. Highly important constructs were considered priorities, particularly when their performance was low, indicating the greatest room for improvement. In contrast, constructs characterized by low importance, but high performance were considered potential areas from which resources could be reallocated to higher-priority constructs.

Finally, we evaluated the model’s in-sample explanatory power using the adjusted coefficient of determination (adjusted *R*^²^). We then assessed its out-of-sample predictive power using the PLSpredict procedure, defined as the model’s ability to generate accurate predictions for holdout-sample observations not used in model estimation [43]. Specifically, we applied a 10-fold cross-validation procedure with 10 repetitions [44]. We further compared the root mean square error (RMSE) and mean absolute error (MAE) of the PLS-SEM model with those of the naive linear model benchmark.

### Ethical Considerations

The study design was approved by the Ethics Committee of the University of Applied Science Wiener Neustadt (September 2021), the Comité national d’éthique de recherche in Luxembourg (202209/07 version 4.0), and the Ministère de la Santé in Luxembourg (840xe52b7). All participants received written information regarding the purpose, potential benefits, and risks of the study and were assured that all data would remain confidential and processed in line with the General Data Protection Regulation. All participants provided electronic consent based on the information provided and voluntarily agreed to participate in the study. All participants had the option to decline participation or withdraw from the study at any time. Participation was voluntary. No monetary or nonmonetary incentives were provided. However, survey participation was allowed during work hours. Participant information was pseudonymized, and access to the data was restricted to the research team.

## Results

### Sample Characteristics

In total, 337 care and nursing staff completed the online survey, including 55 care experts. Of these, 139 (41%) reported using Remote Care Assist at least once per month and were therefore included in the technology acceptance analysis. Among these 139 eligible participants, 45 (32%) were care experts (28 from Austria and 17 from Luxembourg) and 94 (68%) were home care staff (75 from Austria and 19 from Luxembourg).

The sample consisted predominantly of women: 123 (89%) were women, 14 (10%) were men, and 2 (1%) did not disclose their gender. The sample reflected the gender composition of the care workforce in typical care settings [45]. About half of the participants (n=75, 54%) were aged 20-45 years, 62 (45%) were aged 46 years or older, and 2 (1%) did not disclose their age.

### Behavioral Intention to Use Remote Care Assist

Overall, 113 (81%) participants reported a positive “Behavioral Intention to Use” Remote Care Assist (Likert scores 4‐6: somewhat agree, mostly agree, and strongly agree). Of these, 44 (32%) selected “somewhat agree,” 34 (25%) “mostly agree,” and 35 (25%) “strongly agree.” In contrast, 26 (19%) participants reported a negative “Behavioral Intention to Use” Remote Care Assist (Likert scores 1‐3: strongly disagree, mostly disagree, and somewhat disagree). Of these, 6 (4%) selected “strongly disagree,” 5 (4%) “mostly disagree,” and 15 (11%) “somewhat disagree.” The odds of agreement vs disagreement were 4.35:1.

### Measurement Model Evaluation

The measurement model was evaluated for indicator reliability, internal consistency reliability, convergent validity, and discriminant validity. We first evaluated the initial measurement model. Due to high internal consistency estimates (Cronbach α and composite reliability values >0.95) and concerns regarding discriminant validity, particularly between “EBC” and “Behavioral Intention to Use” Remote Care Assist (HTMT ratio=0.884), we reassessed redundancy and content validity, resulting in the removal of 4 redundant items: BITU_2, BITU_3, PE_3, and EBC_2. Consistent with prior studies [46-51] and following parsimonious, pilot-oriented item reduction, “Behavioral Intention to Use” was measured as a single-item construct. For a more detailed description of the variable selection, refer to Multimedia Appendix 1.

In the final measurement model, all multi-item constructs showed strong internal consistency, with Cronbach α values ranging from 0.903 to 0.941. All indicator loadings exceeded the recommended threshold of 0.708 and were statistically significant, supporting indicator reliability. Average variance extracted values were above 0.5 for all multi-item constructs, indicating adequate convergent validity. Discriminant validity was also supported, as all HTMT values (Multimedia Appendix 2) were below the conservative threshold of 0.85 [52] and the bootstrapped HTMT upper 95% CI did not include 1 [31]. Thus, all measurement models were found to be reliable and valid. Multimedia Appendix 3 summarizes the evaluation results for both the proposed model and the final model.

### Structural Model Evaluation and Hypothesis Testing

Based on the achieved sample size of 139 participants, the PLS-SEM power analysis (80% power, *α*=.05) indicated a minimum detectable effect size of 0.211 [32]. VIFs indicated no problematic multicollinearity. All VIFs were below the commonly used threshold of 3, except for the path from “PU” for care staff to “Behavioral Intention to Use” Remote Care Assist (VIF=3.060), which remained below the more liberal threshold of 5 [53] (Multimedia Appendix 4).

**Figure 2.**
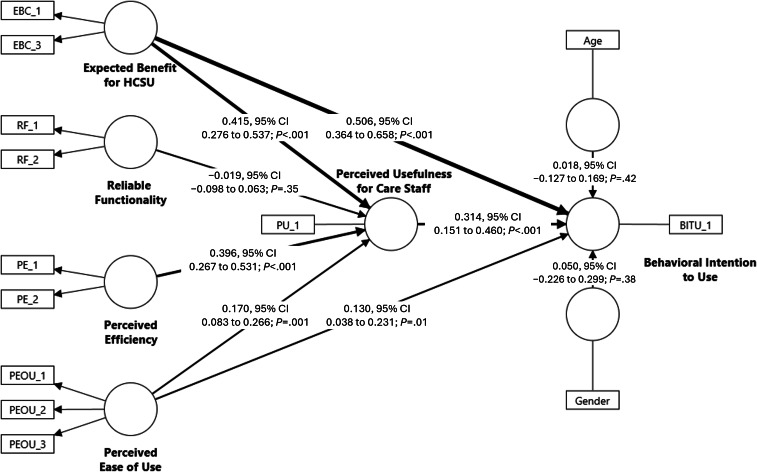
Structural equation modeling results. EBC_1-3: measurement items for “Expected Benefit for HCSU,” RF_1-2: measurement items for “Reliable Functionality,” PE_1-2: measurement items for “Perceived Efficiency,” “PEOU_1-3: measurement items for “Perceived Ease of Use,” PU_1: measurement item for “Perceived Usefulness for Care Staff,” and BITU_1: measurement item for “Behavioral Intention to Use.” HCSU: home care service user.

Figure 2 presents the structural model results. “Behavioral Intention to Use” the Remote Care Assist was positively associated with “EBC” (*β*=0.506, 95% CI 0.364 to 0.658; *P*<.001), “PU” for care staff (*β*=0.314, 95% CI 0.151 to 0.460; *P*<.001), and “PEOU” (*β*=0.130, 95% CI 0.038 to 0.231; *P*=.01), which supported Hypotheses 1, 2a, and 5a. Among all predictors, “EBC” showed the strongest direct association with “Behavioral Intention to Use.”

According to our expectations, “PU” of the Remote Care Assist for care staff was positively associated with “EBC” (*β*=0.415, 95% CI 0.276 to 0.537; *P*<.001), “PE” (*β*=0.396, 95% CI 0.267 to 0.531; *P*<.001), and “PEOU” (*β*=0.170, 95% CI 0.083 to 0.266; *P*=.001), which confirmed Hypotheses 2b, 3, and 5b. In contrast, we found no evidence for Hypothesis 4, as “RF” was not significantly associated with “PU” for care staff (*β*=−0.019, 95% CI −0.098 to 0.063; *P*=.35).

In addition to the direct relationship between “EBC” and “Behavioral Intention to Use,” the association between “EBC” and “Behavioral Intention to Use” Remote Care Assist among care staff was also positively mediated by “PU” for care staff (*β*=0.130, 95% CI 0.061 to 0.194; *P*=.001), supporting Hypothesis 2c. These findings support the hypothesized dual role of “EBC” in shaping care staff’s “Behavioral Intention to Use” Remote Care Assist. On the one hand, care and nursing staff appeared to be guided by a sense of responsibility toward care recipients, as their expectation that Remote Care Assist would benefit home care service users positively contributed to technology acceptance. On the other hand, higher expected benefits for home care service users were associated with greater PU among care and nursing staff, which in turn was positively associated with technology acceptance.

In addition, the association between “PEOU” and “Behavioral Intention to Use” Remote Care Assist among care staff was positively mediated by “PU” (*β*=0.053, 95% CI 0.017 to 0.101; *P*=.02), supporting Hypothesis 5c. The result confirms that easy-to-use apps are perceived as more useful, which in turn increases the acceptance of the technology. Because both the direct and indirect associations were statistically significant and pointed in the same direction, both mediation patterns can be interpreted as complementary mediation [33]. Overall, the results supported Hypotheses 1, 2a, 2b, 2c, 3, 5a, 5b, and 5c, whereas Hypothesis 4 was not supported (Table 1).

Finally, age (*β*=0.018, 95% CI −0.127 to 0.169; *P*=.42) and gender (*β*=0.050, 95% CI −0.226 to 0.299; *P*=.38) as control variables showed no statistically significant associations with “Behavioral Intention to Use” Remote Care Assist.

**Table 1.** Evaluation of path coefficients from the structural equation model and results from hypothesis testing.

Hypothesis	Path	Estimate β (95% CI)	SE	*P* value	Supported
H1	PU^a^ → BITU^b^	0.314 (0.151 to 0.460)	0.094	<.001	Yes
H2a	EBC^c^ → BITU	0.506 (0.364 to 0.658)	0.089	<.001	Yes
H2b	EBC → PU	0.415 (0.276 to 0.537)	0.079	<.001	Yes
H2c	EBC → PU → BITU	0.130 (0.061 to 0.194)	0.041	.001	Yes
H3	PE^d^ → PU	0.396 (0.267 to 0.531)	0.080	<.001	Yes
H4	RF^e^ → PU	–0.019 (–0.098 to 0.063)	0.038	.35	No
H5a	PEOU^f^ → BITU	0.130 (0.038 to 0.231)	0.059	.01	Yes
H5b	PEOU[Table-fn T1_FN6] → PU	0.170 (0.083 to 0.266)	0.056	.001	Yes
H5c	PEOU → PU → BITU	0.053 (0.017 to 0.101)	0.026	.02	Yes

a PU: Perceived Usefulness.

b BITU: Behavioral Intention to Use.

c EBC: Expected Benefit for Home Care Service Users.

d PE: Perceived Efficiency.

e RF: Reliable Functionality.

f PEOU: Perceived Ease of Use.

### Importance-Performance Map Analysis

**Figure 3.**
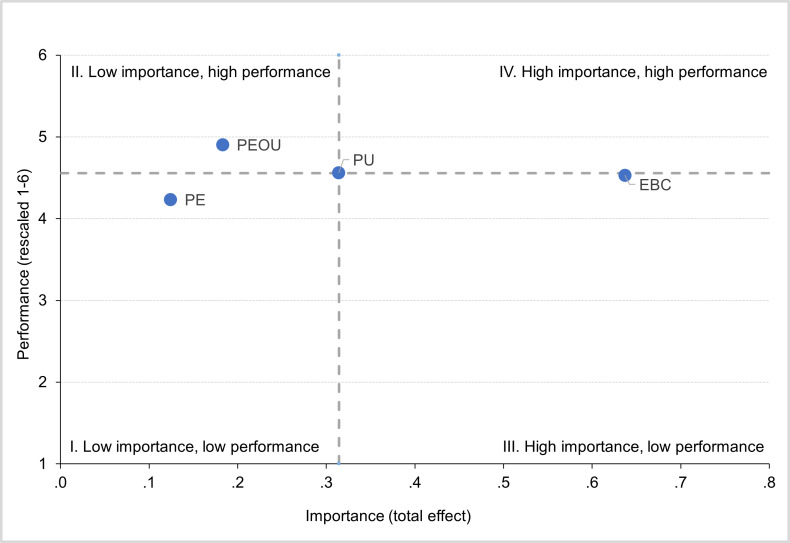
Importance-performance map analysis (IPMA) for the predictors of “Behavioral Intention to Use” Remote Care Assist. EBC: Expected Benefit for Home Care Service Users; PE: Perceived Efficiency; PEOU: Perceived Ease of Use; and PU: Perceived Usefulness.

Figure 3 shows the importance-performance map analysis for the predictors of “Behavioral Intention to Use.” The mean importance threshold, defined as the average total effect across the key constructs, was 0.315. The mean performance threshold, defined as the grand mean of the latent variable scores, was 4.557. The predictors differed in both importance and performance. “EBC” (importance=0.637; performance=4.531) was positioned in the high-importance/low-performance quadrant and emerged as the strongest predictor overall. “PU” (importance=0.314; performance=4.561) and “PEOU” (importance=0.183; performance=4.904) were in the low-importance/high-performance quadrant, indicating these constructs as secondary targets for further optimization. In contrast, “PE” (importance=0.124; performance=4.233) fell within the low-importance/low-performance quadrant and was the least influential predictor.

### Predictive Performance Assessment

The model showed strong explanatory power. Adjusted *R*² values were 0.724 for “PU” for care staff and 0.726 for “Behavioral Intention to Use” Remote Care Assist. These results were slightly above the coefficient of determination compared to the UTAUT [12]. Out-of-sample predictive performance was also supported [43]. The Q²_predict_ values were positive for both constructs (“PU”: 0.716 and “Behavioral Intention to Use”: 0.687), indicating that the PLS-SEM model outperformed the naive indicator-average benchmark [31]. In addition, prediction errors for the PLS-SEM model were lower than those for the naive linear benchmark model. For “PU” and “Behavioral Intention to Use,” the PLS-SEM model yielded lower RMSE (ie, 0.664 and 0.736, respectively) than the benchmark model (RMSE=0.671 and RMSE=0.773). The same pattern was observed for MAE=0.486 and MAE=0.507 vs MAE=0.489 and MAE=0.545, respectively. The lower RMSE and MAE values in the PLS-SEM analysis demonstrated that our model provided more accurate predictions than the naive linear benchmark model, confirming its high predictive power. In summary, the final model demonstrated excellent in-sample predictive power and high out-of-sample predictive power, enabling forecasts of the “Behavioral Intention to Use” Remote Care Assist among care staff.

## Discussion

### Principal Findings

This study tested a contextualized TAM for a newly developed digital communication tool for home care and nursing staff that enabled support and advice via video calls, called Remote Care Assist. Technology acceptance was measured by the “Behavioral Intention to Use” the system among care staff who had already used the system for several months in practice. The most important finding was that a new aspect of technology acceptance, the “EBC,” was the strongest predictor of “Behavioral Intention to Use” the Remote Care Assist. This association was observed both directly and indirectly through “PU” for care staff. These findings suggest that technology acceptance in professional home care is shaped not only by care staff’s expectations of personal or work-related utility, but also by their expectations of the technology’s benefits for home care service users.

The results extend established acceptance theory, building on rational choice [11], by highlighting a relational dimension of technology acceptance in care work. We consider the social influence of home care service users not as a kind of external pressure from other people who want to persuade care staff to perform a behavior, but as an internal perception of care staff that promotes technology acceptance in long-term care settings. In other words, care staff may be more willing to adopt such digital technologies when they believe that they will benefit people receiving care. In addition, this perceived benefit for home care service users may also strengthen their own perception that the technology is useful in practice. Future research could build on these findings and extend them to focus on whether care staff’s emotional attachment to their home care service users or their own standards of professionalism explain why they consider the interests of those in need of care when deciding to accept or reject a particular technology.

Our analysis revealed “PU” as a central and multifaceted predictor and mediator of user acceptance, as supported by previous research [27]. “PU” for care staff was positively associated with care staff’s intention to use Remote Care Assist. “PU” for care staff mediated the associations of both “PEOU” and “EBC” with “Behavioral Intention to Use” the Remote Care Assist.

In addition, the study reaffirmed the relevance of “PEOU” as a positive predictor of “Behavioral Intention to Use” [54,55]. “PEOU” was associated with acceptance both directly and indirectly via “PU,” consistent with established acceptance theory [8,20,38]. These findings indicate that user-friendly digital technologies are more likely to be perceived as useful because they simplify tasks and workflows, which in turn may increase care staff’s willingness to integrate them into daily practice.

Our study confirmed “PE” as an antecedent of “PU” for care staff. The predictor encompasses factors related to improving workflow, speed, and work convenience. By contrast, we found no statistically significant association between “RF” and “PU” for care staff. This nonsignificant result aligns with similar findings in the literature, where the predictor “insecurity,” referring to distrust in telehealth systems’ ability to function well and properly, also did not significantly affect the outcome [56]. A possible explanation for this result is that the Remote Care Assist technology was tested in an exploratory pilot study. Participants in the trial may have been more tolerant of errors and technological issues. However, once Remote Care Assist technology is fully integrated into routine home care practice, users may be less forgiving of technical problems, which could lead to different acceptance outcomes along the implementation process.

Consistent with previous studies [57,58] and in contrast to others who found age [59] and gender [60] to moderate user acceptance, our findings show that the covariates age [55,60] and gender were not statistically significantly associated with the “Behavioral Intention to Use” Remote Care Assist.

Research on technology acceptance provides valuable insights into the potential predictors of care staff’s future intention to use Remote Care Assist or similar digital technologies in care settings. These insights are crucial for management decision-making regarding whether to promote and support technology within a care organization or discontinue its use if it is not well received by end users. For instance, if a predictor of technology acceptance demonstrates low performance but high importance, management should focus on targeted improvements to this predictor. Conversely, if a predictor appears both underperforming and of low importance, it may be appropriate to neglect it and reallocate resources to more promising ones.

### Limitations

Several limitations should be considered when interpreting these findings. First, this was an exploratory pilot study, and the analysis was restricted to participants who reported using the Remote Care Assist system at least once per month. As a result, nonusers and less frequent users were not represented, limiting the generalizability of the findings. Second, the role of the “EBC” was examined for the first time in long-term care settings. Although the findings indicate that this construct may be relevant, further research is needed to establish its conceptual and psychometric validity and to clarify its role in TAMs in care settings. Third, the small sample size did not allow for more detailed multigroup analyses by country or professional role, including direct comparisons between Austria and Luxembourg. Moreover, the cross-sectional design precludes causal inference, and the observed associations should not be interpreted as evidence of temporal or causal relationships. Fourth, the analyses were conducted at the individual level and did not account for potential clustering within care units or organizations. If responses were correlated within units, SEs may have been underestimated, leading to overly narrow CIs and optimistic *P* values. Fifth, “PU” and “Behavioral Intention to Use” were modeled as single-item constructs. Although this may be acceptable in parsimonious or pilot research, single-item measures may increase measurement error and may not fully capture the underlying constructs. Sixth, because predictors and outcomes were collected from the same respondents in a single survey, common method bias cannot be excluded. Future research should include nonusers, use larger samples, further validate the newly introduced construct, and, where feasible, apply longitudinal, multi-item, and multilevel designs.

### Conclusion

Our findings indicate that care organizations intending to implement digital technologies should prioritize utility for both groups: care and nursing staff and their home care service users. Three factors were positively associated with acceptance of telecare technologies: PU, PEOU, and EBC. In their adoption decisions, care staff appear to consider both their own work-related benefit and the expected benefits for the people receiving care. For applications with a clear EBC, this benefit should be explicitly communicated and clearly justified during the implementation process. In contrast, reliability in the sense of flawless functionality of Remote Care Assist was not statistically significantly associated with user acceptance during the pilot implementation phase. Overall, understanding the antecedents of user acceptance among decision-makers can improve the implementation process and the user acceptance of digital applications in long-term care settings, thereby contributing to a better understanding of crucial factors for a successful digitalization of this field.

## Supplementary material

10.2196/80514 Multimedia Appendix 1 Construct definitions and item wording (English, German, and French).

10.2196/80514 Multimedia Appendix 2 Heterotrait-Monotrait ratio of correlations (HTMT) matrix.

10.2196/80514 Multimedia Appendix 3 Evaluation results of the proposed and final measurement models.

10.2196/80514 Multimedia Appendix 4 Variance inflation factor (VIF) statistics.

10.2196/80514 Checklist 1 CHERRIES checklist.

## Abbreviations

CHERRIES: Checklist for Reporting Results of Internet E-Surveys
EBC: Expected Benefit for Home Care Service Users
HCSU: home care service users
HTMT: Heterotrait-Monotrait ratio of correlations
IMP: construct importance
MAE: mean absolute error
PE: Perceived Efficiency
PEOU: Perceived Ease of Use
PERF: construct performance
PLS-SEM: partial least squares structural equation modeling
PU: Perceived Usefulness
RF: Reliable Functionality
RMSE: root mean square error
TAM: Technology Acceptance Model
TE: construct total effect
UTAUT: Unified Theory of Acceptance and Use of Technology
VIF: variance inflation factor
