# Supplementary material for: User Acceptance of Remote Care Assist, a Telecare System for Home Care Among Care and Nursing Staff: Cross-Sectional Pilot Study
Source: JMIR Rehabil Assist Technol. 2026 Jun 3;13:e80514. doi: 10.2196/80514 (PMC13232914; doi:10.2196/80514)
Supplement: Multimedia Appendix 1 [file rehab-v13-e80514-s001.docx]

| Construct | | | Definition | | Description | |
| --- | --- | --- | --- | --- | --- | --- |
| ‘Perceived Usefulness for Care Staff’ | | | An individual’s belief regarding the usefulness of Remote Care Assist. | | Captures the care staff’s perception of Remote Care Assist technology regarding their professional workflow. | |
|  | Item ID | Item (EN) | | Item (DE) | | Item (FR) |
|  | PU_1^a^ | The Remote Care Assist is useful for my job. | | Die Fernunterstützung ist nützlich für meinen Job. | | Le support à distance est utile pour mon travail. |
| Construct | | | Definition | | Description | |
| ‘Expected Benefit for Home Care Service Users’ | | | The degree to which care staff believe that their clients will benefit from using Remote Care Assist technology. | | Assesses the expected benefit that the Remote Care Assist technology provides to home care service users. | |
|  | Item | Item eng^a^ | | Item de^b^ | | Item fr^c^ |
|  | EBC_1^b^ | I think Remote Care Assist is useful for my clients. | | Ich denke, die Fernunterstützung ist nützlich für unsere Klient*innen. | | Je pense que le support à distance est utile pour nos client-e-s. |
|  | EBC_2 | Remote Care Assist supports my clients | | Die Fernunterstützung unterstützt unsere Klient*innen. | | Le support à distance soutient nos client-e-s. |
|  | EBC_3 | Remote Care Assist offers benefits for my clients. | | Die Fernunterstützung bringt für unsere Klient*innen Vorteile. | | Le support à distance présente des avantages pour nos client-e-s. |
| Construct | | | Definition | | Description | |
| ‘Perceived Efficiency’ | | | The degree to which the use of Remote Care Assist will increase the productivity of care staff. | | Focuses on the care staff’s perceptions of how Remote Care Assist technology contributes to more work efficiency. | |
|  | Item ID | Item (EN) | | Item (DE) | | Item (FR) |
|  | PE_1^c^ | Remote Care Assist helps me to accomplish professional tasks faster. | | Die Fernunterstützung hilft mir berufliche Aufgaben schneller zu erledigen. | | Le support à distance m'aide à accomplir des tâches professionnelles plus rapidement. |
|  | PE_2 | Remote Care Assist helps me to accomplish professional tasks more conveniently | | Die Fernunterstützung hilft mir berufliche Aufgaben bequemer zu erledigen. | | Le support à distance m'aide à accomplir des tâches professionnelles plus confortablement. |
|  | PE_3 | Remote Care Assist helps me to accomplish professional tasks more easily. | | Die Fernunterstützung hilft mir berufliche Aufgaben einfacher zu erledigen. | | Le support à distance m'aide à accomplir des tâches professionnelles plus facilement. |
| Construct | | | Definition | | Description | |
| ‘Reliable Functionality’ | | | The degree to which Remote Care Assist is considered to work consistently and error-free in everyday professional use. | | Assesses the perceived reliability and smooth operation of the Remote Care Assist technology. | |
|  | Item ID | Item (EN) | | Item (DE) | | Item (FR) |
|  | RF_1^d^ | Remote Care Assist works flawlessly. | | Die Fernunterstützung funktioniert fehlerfrei. | | Le support à distance fonctionne sans problème. |
|  | RF_2 | Remote Care Assist works reliably. | | Die Fernunterstützung funktioniert zuverlässig. | | Le support à distance fonctionne de manière fiable. |
| Construct | | | Definition | | Description | |
| ‘Perceived Ease of Use’ | | | The degree to which the care staff expects Remote Care Assist technology to be free of effort. | | Assessed how easy the respondents found it to learn and use the Remote Care Assist technology. | |
|  | Item ID | Item (EN) | | Item (DE) | | Item (FR) |
|  | PEOU_1^e^ | Learning how to use Remote Care Assist is easy for me. | | Es fällt/fiel mir leicht, den Umgang mit der Fernunterstützung zu erlernen. | | Il est/était facile pour moi d'apprendre à utiliser le support à distance. |
|  | PEOU_2 | I find Remote Care Assist easy to use. | | Ich finde die Fernunterstützung einfach zu bedienen. | | Je trouve le support à distance facile à utiliser. |
|  | PEOU_3 | I feel able to use Remote Care Assist. | | Ich fühle mich in der Lage die Fernunterstützung zu nutzen. | | Je me sens capable d'utiliser le support à distance. |
| Construct | | | Definition | | Description | |
| ‘Behavioral Intention to Use Remote Care Assist’ | | | The care staff’s subjective probability of using Remote Care Assist in the future. | | Measures the overall acceptance of Remote Care Assist technology. | |
|  | Item ID | Item (EN) | | Item (DE) | | Item (FR) |
|  | BITU_1^f^ | I intend to continue using Remote Care Assist in the future. | | Ich möchte die Fernunterstützung auch zukünftig nutzen. | | Je souhaite continuer à utiliser le support à distance à l'avenir. |
|  | BITU_2 | I would like to continue using Remote Care Assist to communicate with my colleagues. | | Ich möchte weiterhin die Fernunterstützung für die Kommunikation mit meinen Kolleg*innen nutzen können. | | Je souhaite pouvoir continuer à utiliser le support à distance pour communiquer avec mes collègues. |
|  | BITU_3 | I would like the care organisation to continue offering Remote Care Assist. | | Ich möchte, dass die St­ëftung Hëllef Doheem die Fernunterstützung weiterhin zur Verfügung stellt. | | Je souhaite que la Stëftung Hëllef Doheem continue à fournir un support à distance. |

^a^ PU: Perceived Usefulness for Care Staff

^b^ EBC: Expected Benefit for Home Care Service Users

^c^ PE: Perceived Efficiency

^d^ RF: Reliable Functionality

^e^ PEOU: Perceived Ease of Use

^f^ BITU: Behavioral Intention to Use
