# Supplementary material for: User Acceptance of Remote Care Assist, a Telecare System for Home Care Among Care and Nursing Staff: Cross-Sectional Pilot Study
Source: JMIR Rehabil Assist Technol. 2026 Jun 3;13:e80514. doi: 10.2196/80514 (PMC13232914; doi:10.2196/80514)
Supplement: Multimedia Appendix 2 [file rehab-v13-e80514-s002.docx]

|  | PU^a^ | EBC^b^ | PE^c^ | RF^d^ | PEOU^e^ |
| --- | --- | --- | --- | --- | --- |
| PU |  |  |  |  |  |
| EBC | 0.821 |  |  |  |  |
| PE | 0.833 | 0.837 |  |  |  |
| RF | 0.468 | 0.574 | 0.526  0.542 | 0.370 |  |
| PEOU | 0.593 | 0.543 |  |  |  |
| BITU^f^ | 0.789 | 0.847 | 0.728 | 0.458 | 0.585 |

^a^ PU: Perceived Usefulness for Care Staff

^b^ EBC: Expected Benefit for Home Care Service Users

^c^ PE: Perceived Efficiency

^d^ RF: Reliable Functionality

^e^ PEOU: Perceived Ease of Use

^f^ BITU: Behavioral Intention to Use
