# Supplementary material for: User Acceptance of Remote Care Assist, a Telecare System for Home Care Among Care and Nursing Staff: Cross-Sectional Pilot Study
Source: JMIR Rehabil Assist Technol. 2026 Jun 3;13:e80514. doi: 10.2196/80514 (PMC13232914; doi:10.2196/80514)
Supplement: Multimedia Appendix 3 [file rehab-v13-e80514-s003.docx]

| **Construct** | **Items^g^** | **Items^h^** | **Cα ^i^** | **Cα ^j^** | **Rho_a^k^** | **Rho_a^l^** | **AVE ^m^** | **AVE^n^** |
| --- | --- | --- | --- | --- | --- | --- | --- | --- |
| PU^a^ | PU_1 | PU_1 | - | | | | | |
| EBC^b^ | EBC_1 | EBC_1 |  |  |  |  |  |  |
|  | EBC_2 | - | 0.964 | 0.941 | 0.964 | 0.941 | 0.933 | 0.944 |
|  | EBC_3 | EBC_3 |  |  |  |  |  |  |
| PE^c^ | PE_1 | PE_1 |  |  |  |  |  |  |
|  | PE_2 | PE_2 | 0,941 | 0.903 | 0.942 | 0.903 | 0.895 | 0.911 |
|  | PE_3 | - |  |  |  |  |  |  |
| RF^d^ | RF_1 | RF_1 | 0.925 | 0.925 | 0.936 | 0.936 | 0.930 | 0.930 |
|  | RF_2 | RF_2 |  |  |  |  |  |  |
| PEOU^e^ | PEOU_1 | PEOU_1 |  |  |  |  |  |  |
|  | PEOU_2 | PEOU_2 | 0.922 | 0.922 | 0.924 | 0.924 | 0.865 | 0.865 |
|  | PEOU_3 | PEOU_3 |  |  |  |  |  |  |
| BITU^f^ | BITU_1 | BITU_1 |  |  |  |  |  |  |
|  | BITU_2 | - | 0.965 | - | 0.965 | - | 0.977 | - |
|  | BITU_3 | - |  |  |  |  |  |  |

^a^ PU: Perceived Usefulness for Care Staff

^b^ EBC: Expected Benefit for Home Care Service Users

^c^ PE: Perceived Efficiency

^d^ RF: Reliable Functionality

^e^ PEOU: Perceived Ease of Use

^f^ BITU: Behavioral Intention to Use

^g^ Items proposed model

^h^ Items final model

^i^ Cronbach α proposed model

^j^ Cronbach α final model

^k^ Rho_a proposed model

^l^ Rho_a final model

^m^ Average variance extracted proposed model

^n^ Average variance extracted final model
