# Supplementary material for: User Acceptance of Remote Care Assist, a Telecare System for Home Care Among Care and Nursing Staff: Cross-Sectional Pilot Study
Source: JMIR Rehabil Assist Technol. 2026 Jun 3;13:e80514. doi: 10.2196/80514 (PMC13232914; doi:10.2196/80514)
Supplement: Multimedia Appendix 4 [file rehab-v13-e80514-s004.docx]

| Constructs | VIF |
| --- | --- |
| PU^a^ -> BITU^f^ | 3.060 |
| EBC -> PU | 2.812 |
| EBC^b^ -> BITU | 2.815 |
| PE^c^ -> PU | 2.593 |
| RF^d^ -> PU | 1.434 |
| PEOU -> PU | 1.403 |
| PEOU^e^ -> BITU | 1.567 |
| Age-> BITU | 1.075 |
| Gender-> BITU | 1.030 |

^a^ PU: Perceived Usefulness for Care Staff

^b^ EBC: Expected Benefit for Home Care Service Users

^c^ PE: Perceived Efficiency

^d^ RF: Reliable Functionality

^e^ PEOU: Perceived Ease of Use

^f^ BITU: Behavioral Intention to Use
