# Supplementary material for: User Acceptance of Remote Care Assist, a Telecare System for Home Care Among Care and Nursing Staff: Cross-Sectional Pilot Study
Source: JMIR Rehabil Assist Technol. 2026 Jun 3;13:e80514. doi: 10.2196/80514 (PMC13232914; doi:10.2196/80514)
Supplement: Checklist 1 [file rehab-v13-e80514-s005.pdf]

Item Category	Checklist Item	Explanation
Design	Describe survey design	The target population comprised care staff and care experts in Austria and Luxembourg who participated in the ‘Care about Care’ project. The online survey was distributed to all registered project participants. Participation was voluntary.
IRB approval and informed consent process	IRB approval	The study protocol was approved by the ethics board of the University of Applied Sciences Wiener Neustadt (September 2021), the Comité national d’éthique de recherche in Luxembourg (No. 202209/07, Version 4.0), and the Ministère de la Santé in Luxembourg (No. 840xe52b7).
	Informed consent	All participants provided electronic informed consent before participating in the study. The informed consent form included information on the project, purpose/objectives of the digital system to be tested, expected benefits and risks of participation, type of data collected/processed, legal information on data privacy was provided in accordance with GDPR regulation, and contact details of the principal investigator, the contact person in the care organization, and the data protection officer. We also provided information on the survey length. Representatives of the care organizations taking part in the consortium explained the project to their colleagues before obtaining informed consent.
	Data protection	The online survey software LimeSurvey® Version 5.6.68+240625 was hosted by one of the project partners to enhance data protection. Participant-access was controlled via personalized links/unique user IDs. Survey responses were stored with system-generated metadata (unique user IDs, user-token, start language, start date and last-action date). Because token-based access was used, the raw response data were pseudonymized rather than fully anonymous. All project partners signed a joint controller agreement (Art 26 GDPR). Only eligible staff from both research organizations had access to the data.

Development and pre-testing	Development and testing	The technology evaluation survey was developed and programmed using LimeSurvey®. It comprised modules with questions on potential outcomes of the technology, usability, usage behavior and technology acceptance. The results presented in the paper used data from the survey's acceptance module. This module primarily used well-established, validated measurement instruments, which were adapted to the technology being tested. The survey was developed in German. For participants in Luxembourg, a French translation was also provided. Prior to implementation in Austria and Luxembourg, the survey was pretested for usability and technical functionality.
Recruitment process and description of the sample having access to the questionnaire	Open survey vs. Closed survey	The study was conducted as a closed, invitation-only online survey.
	Contact mode	Initial contact was not made online. Eligible participants were informed about the project by their colleagues participating in the project consortium. After consenting to participate, completing a baseline survey, and using the technology for several months, they received a text message containing information and a personalized link to the evaluation survey.
	Advertising the survey	In addition to text-message invitations, participants were informed by project staff about the opportunity to contribute their views on the technology in the evaluation survey.
Survey administration	Web/E-Mail	Participants accessed the survey via a link provided in a text message. The link directed them to a language-specific LimeSurvey® questionnaire. Responses were recorded automatically.
	Context	No website or newsgroup was used for recruitment. Participation was restricted to invited project participants.
	Mandatory/voluntary	Participation was voluntary.
	Incentives	No monetary or non-monetary incentives were provided. However, survey participation was allowed during their work hours.

	Time/date	Trial periods lasted from March–September 2023, with survey data collection from 13 September to 15 October 2023 in Austria and May–October 2023, with survey data collection from 14 September to 30 October 2023 in Luxembourg. Differences in data collection period resulted from a delay in ethical approval in Luxembourg.
	Randomization of items or questions	No randomization was applied, because the response options of the survey required a fixed item order.
	Adaptive questioning	The survey contained questions depending on previous answers, such as the frequency of technology use or experiencing certain situations. However, no conditional programming was required for the technology acceptance section.
	Number of items	The number of items presented per page varied. The survey included 65 questions for care staff and 74 questions for care experts. The technology acceptance section was presented across 6 pages and included 15 items.
	Number of pages	The overall evaluation survey comprised 32 pages for care staff and 39 pages for care experts, including the introduction, informed consent, and debriefing.
	Completeness checks	All items used a forced-response setting. Therefore, the questionnaire could not be continued with missing responses. Participants could discontinue the survey and resume it at any time using their individual token.
	Review step	Respondents were able to review and change their answers, because back navigation was available.
Response rates	Unique site visitor	Not applicable (closed survey).
	View rate (Ratio of unique survey visitors/unique site visitors)	Not applicable (closed survey).
	Participation rate (Ratio of unique visitors who agreed to	444 participants were invited to participate in the survey. 345 initiated the survey, yielding a participation rate of 77.7%.

	participate/unique first survey page visitors)	
	Completion rate (Ratio of users who finished the survey/users who agreed to participate)	Of 345 participants who initiated the survey, 337 completed it, yielding a completion rate of 97.7%.
Preventing multiple entries from the same individual	Cookies used	LimeSurvey® used essential session cookies and Cross-Site Request Forgery (CSRF) cookies. No additional cookies were used.
	IP check	IP addresses were neither used nor stored as part of the survey dataset.
	Log file analysis	Not applicable.
	Registration	Participants received personalized survey links containing unique token. Each token permitted a single complete survey submission only. After a token had been used, repeated access with the same token was blocked. Accordingly, duplicate entries were not possible.
Analysis	Handling of incomplete questionnaires	Responses were included in the analysis if all acceptance items for analysis were available, even if the remainder of the questionnaire was incomplete.
	Questionnaires submitted with an atypical timestamp	A timestamp was set to estimate the time needed to complete the survey. The mean completion time was 20.70 minutes (SD 13.19), and the median was 16.94 minutes (IQR 12.49-23.68), with observed completion times ranging from 7.75 to 79.85 minutes. Although 7 atypical timestamps were identified, they were retained in the analysis because participants could interrupt the survey and resume it at a later time.
	Statistical correction	Not applicable.
